# Comparison of Surface Morphology and Tool Wear in the Machining of Ti-6Al-4V Alloys with Uncoated and TiAlN Tools under Dry, Minimum Quantity Lubrication, Flood Cooling, and Low-Temperature Spray Jet Cooling Conditions

**DOI:** 10.3390/mi14061263

**Published:** 2023-06-17

**Authors:** Jinfu Zhao, Zhanqiang Liu, Zipeng Gong, Annan Liu, Yukui Cai, Bing Wang, Qinghua Song, Xiaoliang Liang, Yanbin Zhang, Zhicheng Zhang, Haiyang Ji

**Affiliations:** 1 School of Mechanical Engineering, Shandong University, Jingshi Road 17923, Jinan 250061, China; sduzhaojinfu@sdu.edu.cn (J.Z.); sduliuannan@mail.sdu.edu.cn (A.L.); caiyukui@sdu.edu.cn (Y.C.); sduwangbing@sdu.edu.cn (B.W.); ssinghua@sdu.edu.cn (Q.S.); sduliangxiaoliang@mail.sdu.edu.cn (X.L.); sduzhangzhicheng@mail.sdu.edu.cn (Z.Z.); sdujihaiyang@mail.sdu.edu.cn (H.J.); 2 Key National Demonstration Center for Experimental Mechanical Engineering Education, Key Laboratory of High Efficiency and Clean Mechanical Manufacture of MQE, Jinan 250061, China; 3 Sinotruk (Jinan) Axle Co., Ltd., Jinan 250061, China; gziwen@126.com; 4 School of Mechanical and Automotive Engineering, Qingdao University of Technology, Qingdao 266520, China; qdlg@163.com; 5 Department of Industrial and Systems Engineering, The Hong Kong Polytechnic University, Hong Kong 999077, China

**Keywords:** TiAlN coating, uncoated carbide tool, Ti-6Al-4V, machined surface roughness, tool life, cooling condition

## Abstract

TiAlN-coated carbide tools have been used to machine Ti-6Al-4V alloys in aviation workshops. However, the effect of TiAlN coating on surface morphology and tool wear in the processing of Ti-6Al-4V alloys under various cooling conditions has not been reported in the public published literature. In our current research, turning experiments of Ti-6Al-4V with uncoated and TiAlN tools under dry, MQL, flood cooling, and cryogenic spray jet cooling conditions were carried out. The machined surface roughness and tool life were selected as the two main quantitative indexes for estimating the effects of TiAlN coating on the cutting performance of Ti-6Al-4V under various cooling conditions. The results showed that TiAlN coating makes it hard to improve the machined surface roughness and tool wear of a cutting titanium alloy at a low speed of 75 m/min compared to that achieved by uncoated tools. The TiAlN tools presented excellent tool life in turning Ti-6Al-4V at a high speed of 150 m/min compared to that achieved by uncoated tools. From the perspective of obtaining finished surface roughness and superior tool life in high-speed turning Ti-6Al-4V, the selection of TiAlN tools is feasible and reasonable under the cryogenic spray jet cooling condition. The dedicative results and conclusions of this research could guide the optimized selection of cutting tools in machining Ti-6Al-4V for the aviation industry.

## 1. Introduction

The Ti-6Al-4V alloy is widely utilized in the aviation industry due to its good properties including low density, high strength-to-weight ratio, and high corrosion resistance [1,2]. In the aviation industry, titanium alloys are commonly used as structural materials and aircraft fuselage skins [3]. However, the Ti-6Al-4V alloy is deemed as the difficult-to-cut material due to its low thermal conductivity of 15.24 W/(m·K), low elasticity modulus 110 GPa, and high chemical reactivity with O_2_, H_2_, and N_2_, etc. The mechanical friction and chemical reaction at tool–chip–workpiece contact interfaces deteriorated due to the induced high cutting temperature rises and cutting forces, which deteriorated the tool machining performance and surface topography [4]. It was valuable to research the improved cutting tool and high-efficiency cooling conditions to overcome this problem.

Recently, extensive research has been utilized to improve the machinability of titanium alloys. For instance, unconventional cooling-lubrication methods like cryogenic cooling and high-pressure emulsion provided extended tool life compared to the dry environment in turning of Ti-6Al-4V using straight carbides. Pervaiz et al. [5] conducted the turning process of Ti-6Al-4V with uncoated carbide tools under dry, MQL, and flood cooling conditions, respectively. The results showed that the uncoated tools exhibited superior cutting performance at low cutting speeds. Recently, Chetan et al. [6] analyzed the machining performance of TiN tools in continuous turning Ti-6Al-4V under dry and MQL cooling conditions. Great improvement in tool wear was obtained under MQL cooling conditions. Choudhary and Paul [7] illustrated that the multilayered TiN/TiCN/TiN tools exhibited better tool life compared to that of TiN tools under flood cooling (FC) conditions. They illustrated that the uncoated carbide tools were suitable for machining Ti-6Al-4V with the comprehensive consideration of specific cutting energy, dimensional deviation, and average principal flank wear.

Physical vapor-deposited (PVD)-coated tools have also been developed for the machining of Ti-6Al-4V. Jaffery and Mativenga [8] compared the tool wear process in machining Ti-6Al-4V within cutting speed ranges of 50–150 m/min by uncoated and various PVD-coated tools, such as NbN, AlCrN, and Ti_6_N tools. The results showed that the Ti_6_N coating improved machining performance during high-speed cutting processes due to the formation of titanium aluminides (Ti_3_Al/Ti_6_Al and TiAlN). They found that the tool coating with suitable thermal properties could improve the machining performance of Ti-6Al-4V alloys. Zeilmann and Weingaertner [9] confirmed the good performance of TiAlN tools compared to the CrCN and TiCN tools in drilling Ti-6Al-4V under MQL cooling conditions. Hao et al. [10,11] found the formation of the Al_2_O_3_ layer in turning the difficult-to-cut materials with PVD TiAlN tools based on the chemical element analysis on the coating cross-section. The thermal conductivity of Al_2_O_3_ was about 6 W/(m·K) at an elevated temperature of 700 °C. The Al_2_O_3_ layer could exhibit excellent thermal barrier effects to protect the tool substrate in the high-speed cutting process.

Several researchers discussed the cutting performances of uncoated and TiAlN tools under dry, spray jet cooling (SJC), and flood cooling (FC) conditions. Pervaiz1 et al. [5] analyzed the cutting forces, tool wear, and surface roughness in turning Ti-6Al-4V alloys under dry, SJC, and flood cooling conditions within the speed ranges of 30–120 m/min. They found that the SJC and flood cooling conditions improved the tool flank wear propagation. The effects of cooling conditions on surface roughness after turning Ti-6Al-4V alloys with uncoated and TiAlN tools were different. Nandgaonkar et al. [12] found that higher lubrication at the tool–chip interface could be obtained in drilling TiAlN tools under MQL cooling conditions. Strano et al. [13] conducted the turning process of Ti-6Al-4V with TiAlN tools under dry and deep cryogenic treatment (DCT) at cutting speeds 40 m/min and 50 m/min. The DCT could improve the TiAlN tool wear resistance at high cutting speed. Liang et al. [14] conducted the turning process of Ti-6Al-4V alloys under dry and high-pressure coolant supplies (HPCS). The results showed that the effects of cooling conditions on tool–chip–workpiece friction characteristics and surface quality were different at varied cutting speeds.

Some newly developed cooling conditions have also been utilized for machining Ti-6Al-4V. Cryogenic cooling conditions such as low-temperature liquid nitrogen and low-temperature CO_2_ were utilized for improving cutting performance in machining Ti-6Al-4V [15,16,17]. The deep cryogenic and high-pressure cooling devices are expensive, and complex compared to the SJC and MQL cooling devices. Nowadays, a low-cost refrigerator is connected to the spray jet cooling system to obtain lower cooling temperatures of atomized fluid droplets [18,19]. Some researchers confirmed that the cryogenic spray jet cooling (CSJC) method could obtain better cutting performance compared to the traditional SJC method. However, the effect of TiAlN coating on machining Ti-6Al-4V under CSJC conditions is not sufficiently analyzed compared to that under other cooling conditions.

In this research, the turning experiments of Ti-6Al-4V under dry, MQL, flood cooling, and CSJC conditions were conducted, respectively. The machined surface roughness and tool life of uncoated and TiAlN tools were selected as the two main quantitative indexes for estimating the coating effects on machining performance. The coating influencing regularities on the machined surface roughness and tool life in turning Ti-6Al-4V under dry and various conditions were obtained compared to that with an uncoated tool. The research results could help to determine suitable tools for machining Ti-6Al-4V under specific cooling conditions.

## 2. Materials and Properties

### 2.1. Workpiece of Ti-6Al-4V

**Figure 1:**
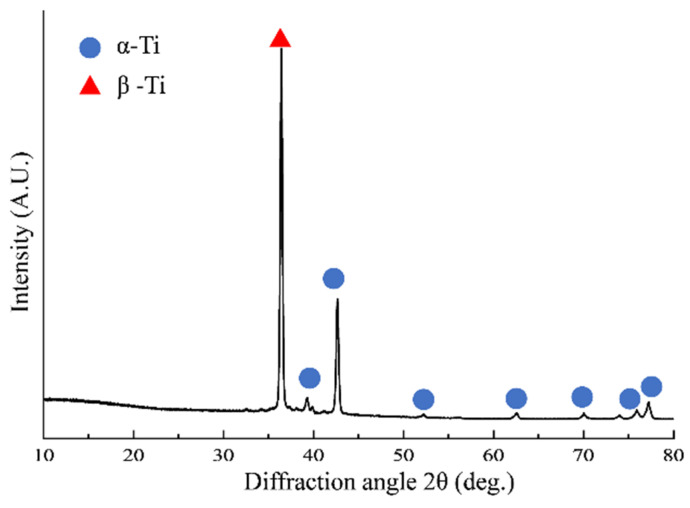
Phase composition of Ti-6Al-4V.

The Ti-6Al-4V alloy was obtained with a duration time of 1–4 h at heat treatment temperature 700–800 °C, which was then cooled to room temperature in the air environment. Figure 1 indicated that the α-phase (volume fraction 91.7%) and β-phase (volume fraction 8.3%) composed the alloy. The chemical element composition, mechanical physical properties of Ti-6Al-4V alloy at room, and evaluated temperatures are obtained in Table 1, Table 2 and Table 3, respectively.

### 2.2. Cutting Tools

**Figure 2:**
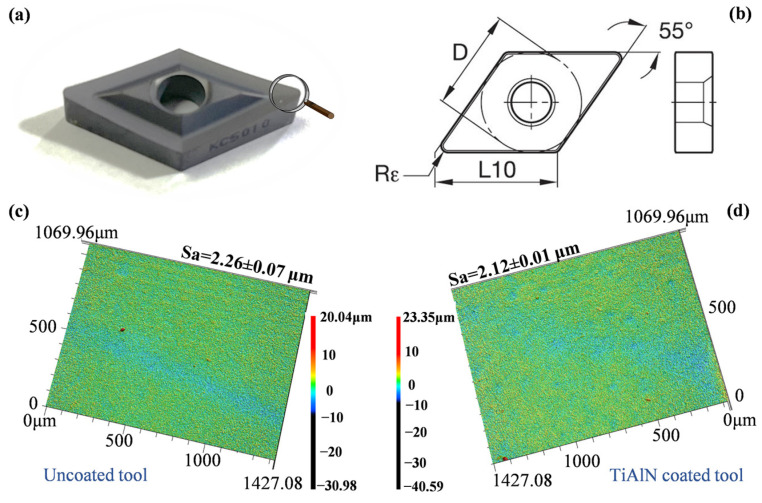
(**a**) Selected TiAlN tool; (**b**) brief schematic of the selected cutting tools. Surface topography and surface roughness of rake face of (**c**) the uncoated tool and (**d**) the TiAlN tool.

The uncoated cemented carbide tool (VNMP160408 K313, Kennametal Inc., Pittsburgh, PA, USA) and PVD TiAlN-coated carbide tool (VNMP160408 KC5010, Kennametal Inc.) were utilized in Figure 2a. The main geometric angles of utilized cutting tools included a diamond angle of 55°, a lead angle of 90°, a side rake angle of 12.8°, a clearance angle of 11°, and a tool edge radius of 0.08 mm as shown in Figure 2b. The surface topographies and surface roughness of uncoated and TiAlN tool rake faces are compared in Figure 2c,d, respectively. The deposited TiAlN coating decreased about 6.19% of the rake face roughness value from Sa = 2.26 μm to Sa = 2.12 μm compared to that of the uncoated tool.

**Figure 3:**
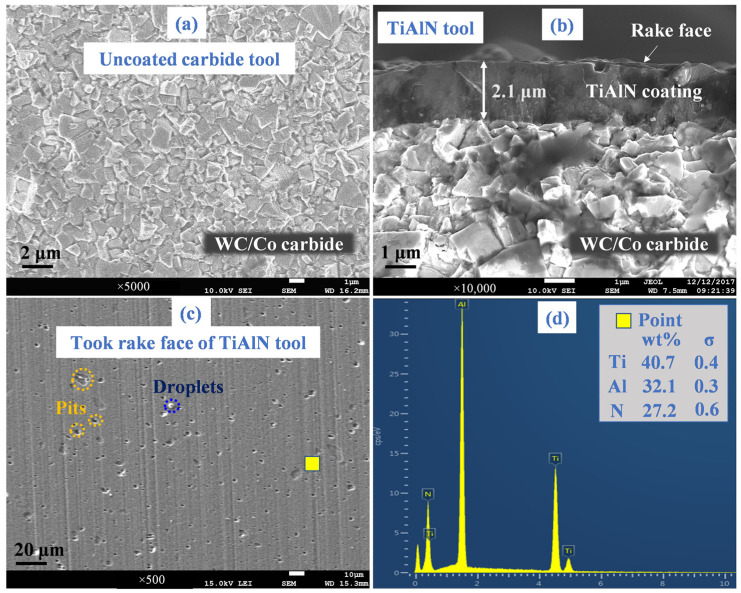
(**a**) Cross-sectional topography of the uncoated tool. TiAlN tool: (**b**) cross-sectional topography; (**c**) surface topography of the rake face; (**d**) coating chemical composition.

Figure 3a,b depicts the cross-sectional topographies of uncoated and TiAlN tools, respectively. The substrate carbide contained 89.5% content of the WC phase and 10.5% content of the Co phase. Figure 3b illustrates that the TiAlN coating thickness was measured as 2.1 μm. The tool rake face topography and coating chemical composition of TiAlN tools are shown in Figure 3c,d, respectively. Some pits and droplets generated during the coating deposition process were detected in Figure 3c. The weight ratio of Ti:Al:N atoms was measured as 40.7:32.1:27.2.

## 3. Experimental Procedure

### 3.1. Turning Process under Various Cooling Conditions

**Figure 4:**
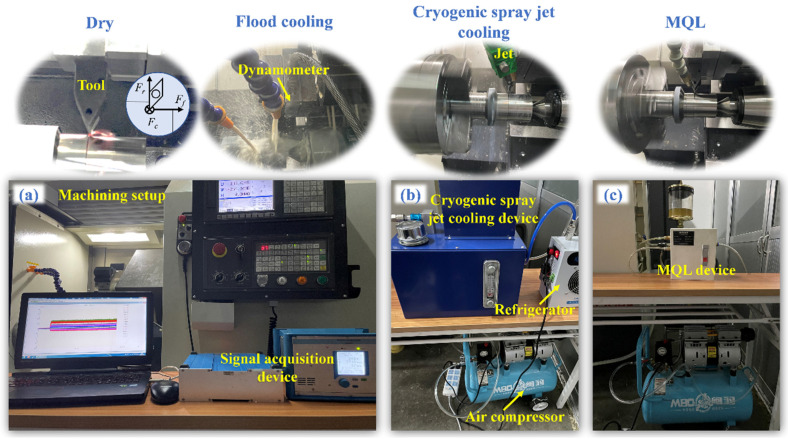
Turning procedure of Ti-6Al-4V under dry and three types of cooling conditions. (**a**) Turning experimental set-up. (**b**) CSJC device. (**c**) MQL device.

The turning experiments of Ti-6Al-4V were conducted in the numerical control lathe of Baoji CK6560A under dry and three types of cooling conditions (Figure 4). The utilized cutting tools were fabricated into the cutter arbor DDNNR2525M15KC06. The cutting speeds were 75 m/min and 150 m/min. The feed rate was 0.1 mm/rev. The depth of cut was 1 mm. The refrigerator was connected to the atomized instrument. The cooling temperature of the atomized fluid droplets was set to 0 °C. The dry and three types of cooling conditions are summarized in Table 4. The angle between the axis of the external jet and the tool flank face was adjusted to 15° in the turning process under CSJC and MQL cooling conditions.

### 3.2. Detection and Characterization

The forces including the tangential force *F_c_*, radial force *F_r_*, and axial force *F_z_* were captured timely with the three-component measuring system with the type of dynamometer being the Kistler 9229A. The average force value and its standard deviation could be calculated based on a 95% confidence level when the cutting process entered the stable stage. The cross-sectional topography and coating chemical element composition could be obtained by a scanning electron microscope (SEM, JSM-6510) coupled with an energy-dispersive X-ray spectrum (EDS). The diffraction peaks of Ti-6Al-4V were captured with the X-ray diffractometry (XRD) spectrum Smartlab.

The surface topography was analyzed with the arithmetical mean height Sa and the developed interfacial area ratio Sdr based on standard ISO 25178-2. The surface topographies, surface roughness Sa, and tool geometric angles could be determined with a shape laser microscope (VK-X250K).

## 4. Results and Discussion

### 4.1. Measured Cutting Forces and Friction Coefficient

The resultant force *F*_∑_ reflected the material deformation resistance during the turning process of Ti-6Al-4V with uncoated and TiAlN tools, which could be determined with Equation (1) [20,21,22]. The average tool–chip contact friction characteristics could be represented by the average tool–chip friction coefficient *μ*, which could be calculated with average tangential force *F_c_*, radial force *F_r_*, axial force *F_z_*, and side rake angle *γ* in Equation (2) [23,24].

(1) $$F_{\Sigma }=\sqrt{F_{c}{}^{2}+F_{r}{}^{2}+F_{z}{}^{2}}$$

(2) $$\mu =\frac{F_{c}\tan\gamma_{0}+\sqrt{F_{r}{}^{2}+F_{z}{}^{2}}}{F_{c}-\sqrt{F_{r}{}^{2}+F_{z}{}^{2}}\tan\gamma_{0}}$$

**Figure 5:**
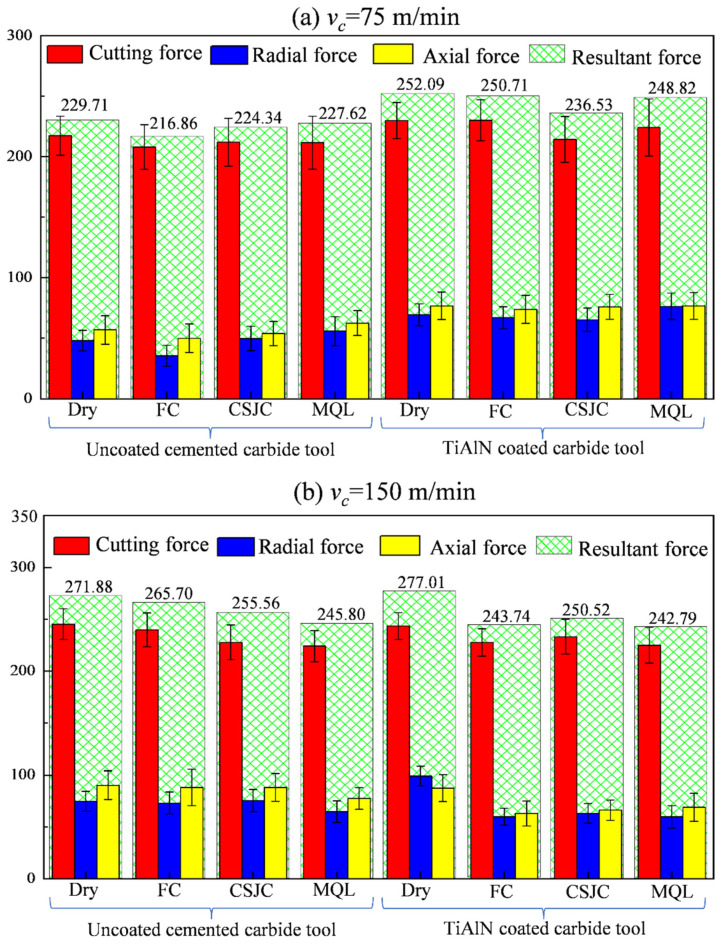
Measured cutting force, radial force, and axial force during the turning process of Ti-6Al-4V under dry and various cooling conditions at different cutting speeds: (**a**) 75 m/min and (**b**) 150 m/min.

**Figure 6:**
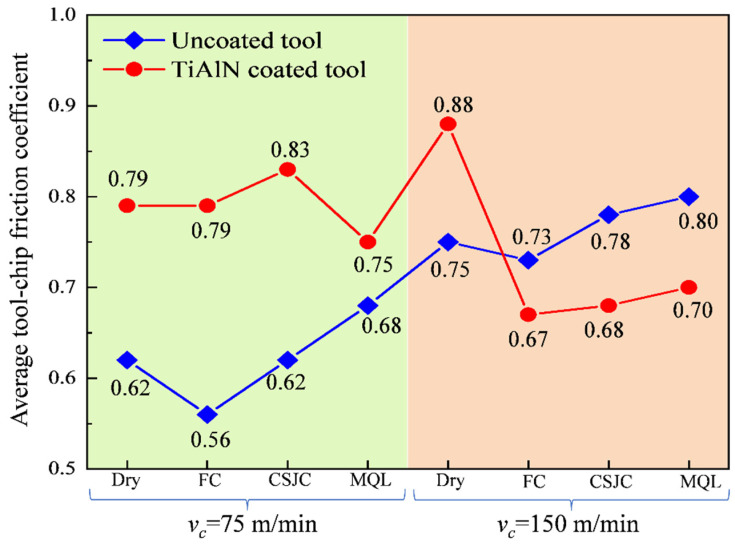
Average tool–chip friction coefficient during the turning process of Ti-6Al-4V under dry and various cooling conditions at cutting speeds of 75 m/min and 150 m/min.

Figure 5a,b depicts the measured cutting force, radial force, axial force, and calculated resultant forces. Figure 6 shows the tool–chip contact friction coefficients during the turning process of Ti-6Al-4V under dry, CF, CSJC, and MQL cooling conditions. The low resultant cutting forces were induced in the dry turning process of Ti-6Al-4V by uncoated carbide tools due to the low friction coefficients. The contact friction characteristics in the dry-turning Ti-6Al-4V alloys could not be improved by the deposited TiAlN coating compared with those achieved by uncoated tools.

Figure 5 shows that the low resultant cutting forces were induced in the turning process of Ti-6Al-4V with uncoated tools under various cooling conditions at a low speed of 75 m/min and that the TiAlN-coated tools could decrease 21.96 N, 5.04 N, and 3.01 N of the resultant cutting forces in turning Ti-6Al-4V with uncoated tools under CF, CSJC, and MQL cooling conditions at a high speed of 150 m/min, respectively. The high cutting speed meant high chip-flowing speed. The high flowing speed of the generated chip could decrease the tool–chip contact time, thus the heat flux could be taken away in time due to coating thermal barrier effects. The TiAlN coating could improve the tool–chip contact friction characteristics in high-speed cutting Ti-6Al-4V alloys compared to those with uncoated tools. The coolant of water-soluble cutting fluid LN6889 exhibited good lubrication and cooling effects on the improvement of friction characteristics at higher speeds.

The TiAlN-coated tools could decrease 8.22%, 12.82%, and 12.50% of the tool–chip contact friction coefficients in dry turning Ti-6Al-4V with uncoated tools under CF, CSJC, and MQL cooling conditions at a high speed of 150 m/min, respectively, as shown in Figure 6. It was illustrated that the micro-droplets of cutting fluid LN6889 and animal oil could be easily dissipated into the tool–chip contact interface between the TiAlN tool and Ti-6Al-4V compared to that with uncoated tools under CSJC and MQL conditions.

### 4.2. Machined Surface Topographies

**Figure 7:**
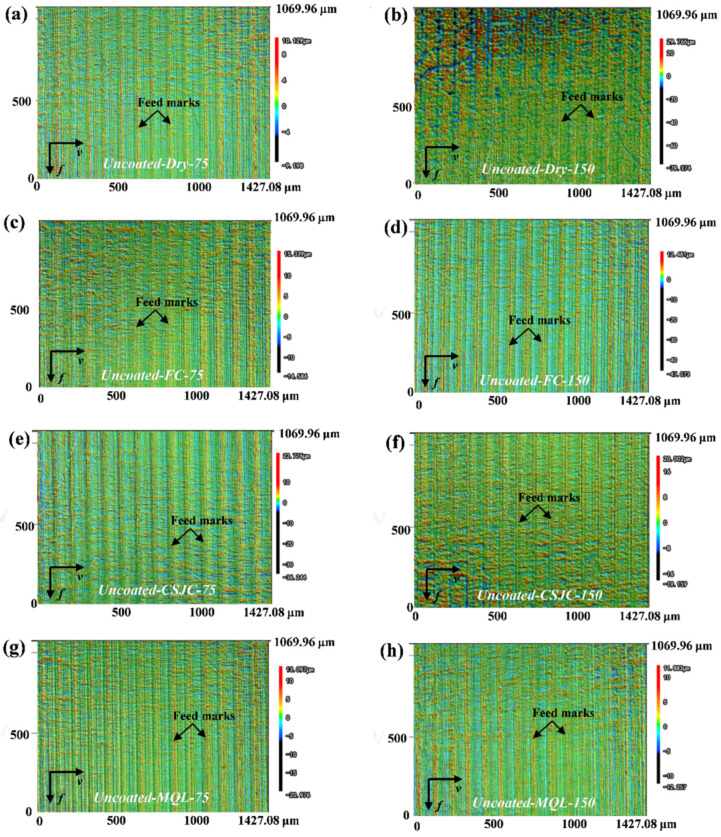
Surface topographies of Ti-6Al-4V after the turning process with uncoated carbide tools under dry and various cooling conditions at cutting speeds of 75 m/min and 150 m/min.

**Figure 8:**
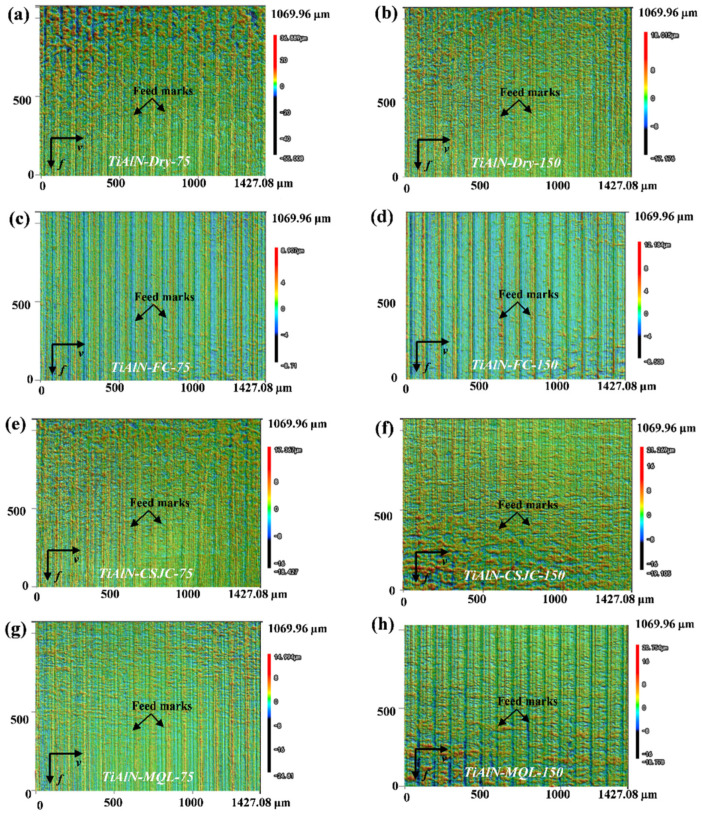
Surface topographies of Ti-6Al-4V after the turning process with TiAlN-coated carbide tools under dry and various cooling conditions at cutting speeds of 75 m/min and 150 m/min.

**Figure 9:**
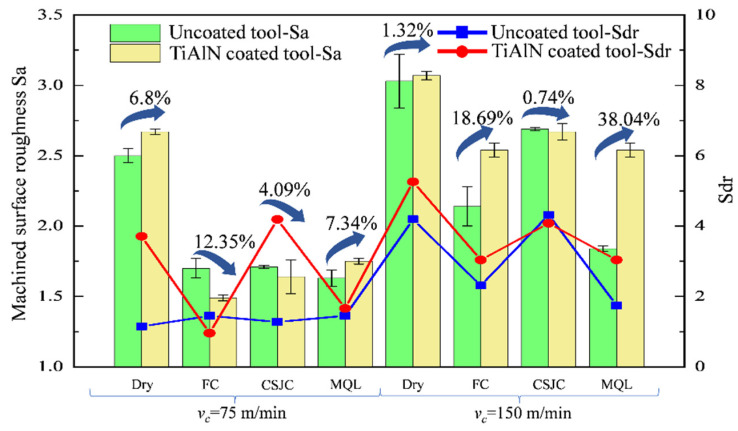
Measured surface roughness of Ti-6Al-4V after the turning process with uncoated and TiAlN tools under dry and various cooling conditions at cutting speeds of 75 m/min and 150 m/min.

Figure 7 and Figure 8 depict the 3D surface topographies of Ti-6Al-4V after the turning process with uncoated tools at various cutting speeds. The evident feedback on the turned surface of Ti-6Al-4V was detected under dry and various cooling conditions. The surface topography parameters Sa and Sdr are presented in Figure 9. The Sa was increased at least 12.88% after initial turning Ti-6Al-4V when the speed increased from 75 m/min to 150 m/min for various cutting couples. It may be illustrated that severe material deformation of the Ti-6Al-4V surface layer could be induced under high-speed cutting speeds.

Figure 9 shows that the various cooling conditions (CF, CSJC, and MQL) improved the surface roughness of Sa by (32.00%, 31.60%, and 34.80%) and (29.37%, 11.22%, and 39.27%), respectively, compared to that under the dry cutting process with uncoated tools at cutting speeds of 75 m/min and 150 m/min. The various cooling conditions (CF, CSJC, and MQL) improved the surface roughness of Sa by (44.19%, 38.58%, and 34.46%) and (17.26%, 13.03%, and 17.26%), respectively, compared with that under the dry cutting process with TiAlN tools at cutting speeds of 75 m/min and 150 m/min. This may illustrate that the lubricants could improve the tool–workpiece contact interface characteristics.

The superior surface roughness of Sa was obtained in turning the Ti-6Al-4V alloy with uncoated and TiAlN tools under CF, CSJC, and MQL cooling conditions compared to that under dry cutting conditions. MQL and FC were suitable cooling conditions in turning Ti-6Al-4V with uncoated and TiAlN tools by obtaining the lowest surface roughness, respectively. The machined surface roughness in turning the Ti-6Al-4V with the TiAlN tool under MQL cooling conditions was similar to that under flood cooling conditions at a high speed of 150 m/min.

Figure 9 shows that the deposited TiAlN coating increased the machined surface roughness in the turning wTi-6Al-4V alloy by 6.80% and 7.34% at a low speed of 75 m/min under dry and MQL cooling conditions. The deposited TiAlN coating increased the machined surface roughness by 1.32%, 18.69%, and 38.04% in turning the Ti-6Al-4V alloy at a high speed of 150 m/min under dry, FC, and MQL cooling conditions, respectively. It may be illustrated that the macro-droplets of lubricant LN6889 were difficult to dissipate into the tool–workpiece contact interface to improve the surface roughness in turning Ti-6Al-4V with TiAlN tools at a high speed of 150 m/min compared to that at a low speed of 75 m/min. The TiAlN coating with relatively low thermal conductivity could prevent more heat flux from dissipating into the workpiece to deteriorate the machined surface topography. The deposited TiAlN coating decreased 4.09% and 0.74% of the machined surface roughness in turning the Ti-6Al-4V alloy at cutting speeds of 75 m/min and 150 m/min under cryogenic spray jet cooling conditions, respectively. It may be illustrated that the micro-droplets of lubricant LN6889 easily dissipated into the tool–workpiece contact interface in turning the Ti-6Al-4V with TiAlN tools compared to that with uncoated tools. The dissipated micro-droplets could take away massive heat and improve the friction characteristics at the tool–workpiece interface to obtain good surface topography.

### 4.3. Tool Wear after Removing Specific Material Volume

**Figure 10:**
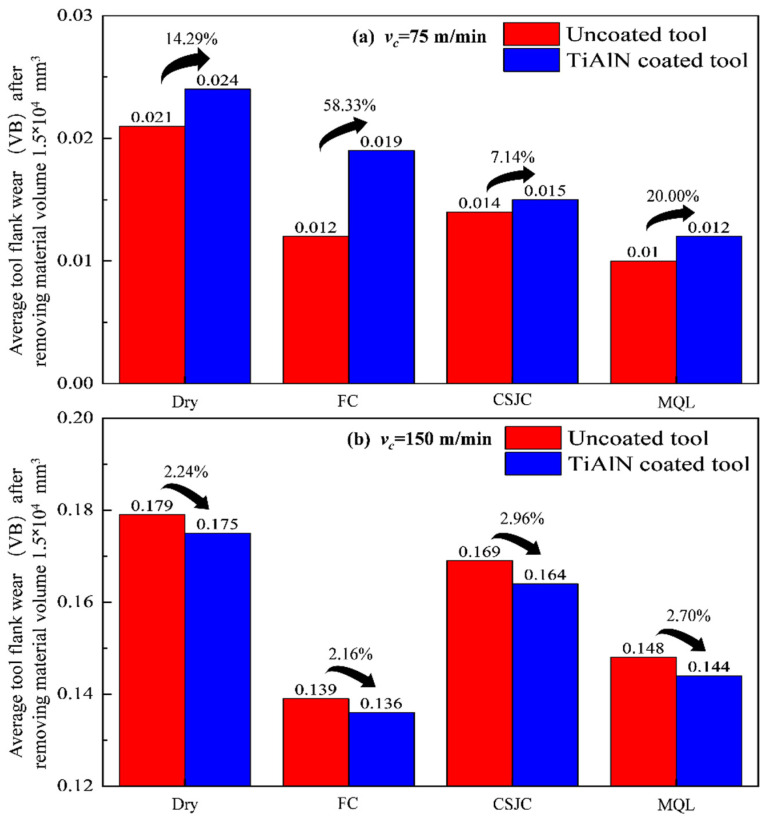
Average tool flank wear (VB) after removing the material volume of 1.5 × 10^4^ mm^3^ of Ti-6Al-4V with uncoated and TiAlN-coated carbide tools under dry and various cooling conditions at cutting speeds of 75 m/min and 150 m/min.

Figure 10 shows the values of average tool flank wear (VB) after removing the material volume of 1.5 × 10^4^ mm^3^ of Ti-6Al-4V with the selected tools under dry and various cooling conditions at cutting speeds of 75 m/min and 150 m/min. The severe tool flank wear could be induced in turning Ti-6Al-4V at high speed compared with that at low speed. The cutting tools faced severe mechanical loads due to the high cutting forces induced in the initial high-speed cutting Ti-6Al-4V. The severe mechanical loads accelerated the tool wear procedure.

The values of VB for TiAlN tools were increased 14.29%, 58.33%, 7.14%, and 20.00% compared with that for uncoated tools under dry, CF, CSJC, and MQL cooling conditions at a cutting speed of 75 m/min, respectively. The values of VB for TiAlN tools could improve by 2.24%, 2.16%, 2.96%, and 2.70% compared with that for uncoated tools under dry, CF, CSJC, and MQL cooling conditions at a high cutting speed of 150 m/min, respectively. This may be related to the fact that the TiAlN coating could not improve the average tool–workpiece contact friction characteristics in turning Ti-6Al-4V alloys compared with that achieved by uncoated tools. The worse friction status could accelerate the tool wear process.

The sequences of VB values for uncoated and TiAlN tools after turning Ti-6Al-4V under various cooling conditions were dry > FC > MQL > CSJC and dry > FC > CSJC > MQL at a low speed of 75 m/min, respectively. The similar sequences of VB values for uncoated and TiAlN tools after turning Ti-6Al-4V under various cooling conditions were dry > CSJC > MQL > FC at a high speed of 150 m/min. The micro-droplets of cutting fluid LN6889 and animal oil could be easily dissipated into the tool–chip contact interface compared to that under FC cooling conditions at a low speed of 75 m/min. The severe tool–workpiece contact characteristics could deteriorate the mechanical and thermal loads applied on cutting tools at a high speed of 150 m/min. The utilization of flood cutting could obtain better cooling effects and improve the tool life compared to that under CSJC and MQL cooling conditions.

**Figure 11:**
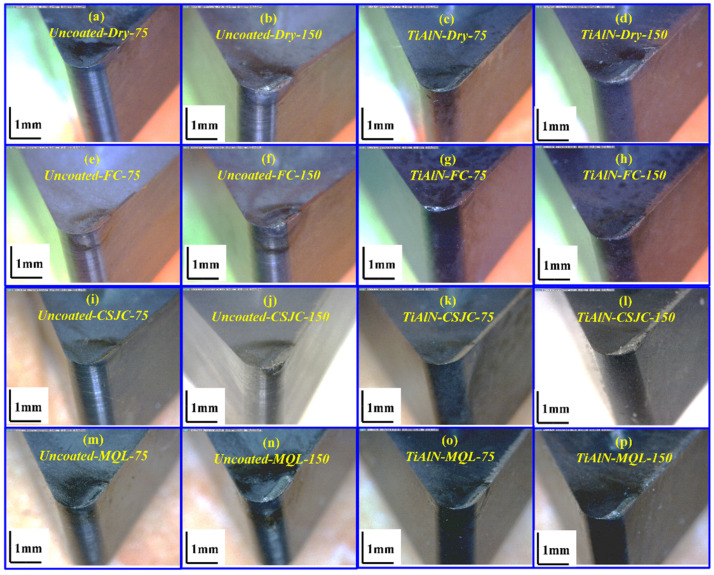
Tool wear topographies after removing the material volume of 1.5 × 10^4^ mm^3^ of Ti-6Al-4V with uncoated and TiAlN tools under dry and various cooling conditions at cutting speeds of 75 and 150 m/min.

Figure 11 illustrates the tool wear topographies after removing the material volume of 1.5 × 10^4^ mm^3^ of Ti-6Al-4V with the selected tools under dry and various cooling conditions at cutting speeds of 75 and 150 m/min. The relatively evident crater and flank wear could be detected after turning Ti-6Al-4V at a higher speed. The average tool flank wear in turning Ti-6Al-4V at a high speed of 150 m/min was detected one order of magnitude higher than that at a low speed of 75 m/min after removing the same volume of workpiece materials.

### 4.4. Tool Life in High-Speed Turning Ti-6Al-4V with Uncoated and TiAlN-Coated Tools

**Figure 12:**
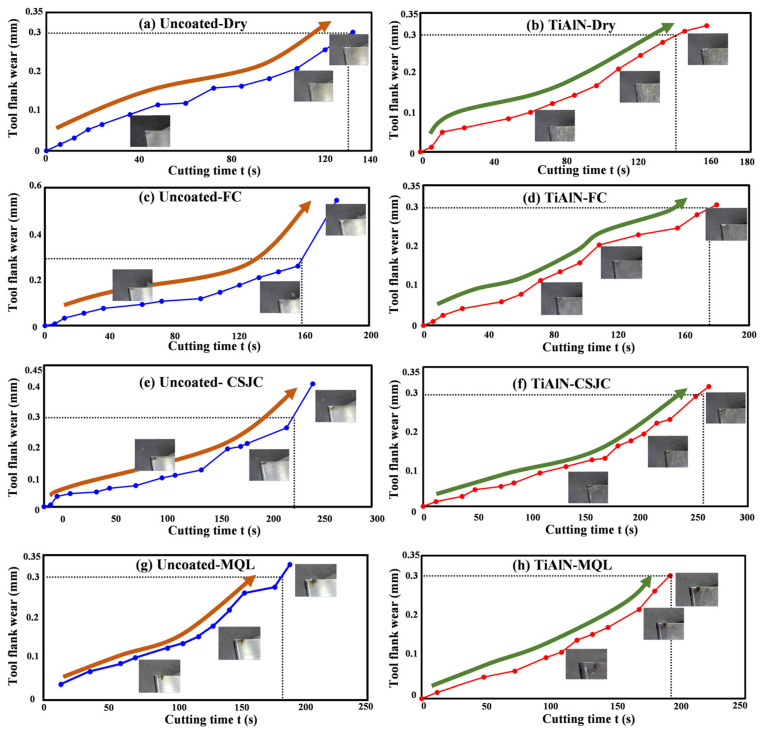
Tool life curves in turning Ti-6Al-4V with uncoated and TiAlN tools under dry and various cooling conditions at a high cutting speed of 150 m/min.

**Figure 13:**
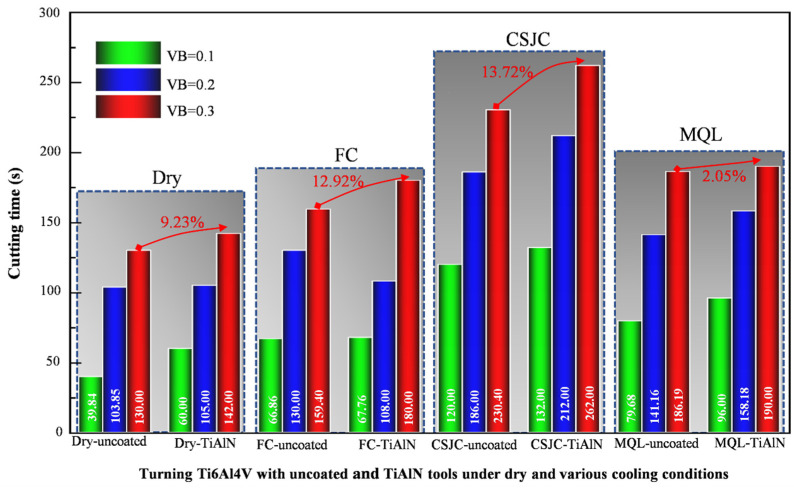
Tool life values when the flank wear VB attained 0.1, 0.2, and 0.3 mm in turning Ti-6Al-4V with uncoated and TiAlN tools under dry and various cooling conditions at a cutting speed of 150 m/min.

Figure 12 depicts the tool life curves in turning Ti-6Al-4V with the selected tools under dry and various cooling conditions at a high cutting speed of 150 m/min. The tool wear processing could be split into three parts based on tool wear rates. The three parts include the initial, normal, and severe wear stages. The corresponding tool life values when the average tool flank wear VB attained 0.1, 0.2, and 0.3 mm are summarized in Figure 13. The cryogenic spray jet cooling exhibited the most superior improvement effects on tool life in high-speed turning Ti-6Al-4V with uncoated and TiAlN tools compared to that under other cooling conditions. The sequence for the improvement effects of various cooling conditions on tool life in high-speed turning Ti-6Al-4V with uncoated and TiAlN tool was CSJC > MQL > FC > dry. It was illustrated that the micro-droplets of the lubricant LN6889 and animal oil could easily dissipate into the tool–chip–workpiece contact interface. The severe friction characteristics at the contact interfaces were improved to decrease the tool wear rate in turning Ti-6Al-4V under CSJC and MQL cooling conditions compared to that under flood cooling conditions.

Figure 13 shows that the deposited TiAlN coating improved the tool life by 9.23%, 12.92%, 13.72%, and 2.05% in high-speed turning Ti-6Al-4V under dry, flood cooling, CSJC, and MQL conditions compared to that achieved by uncoated carbide tools, respectively. The improvement effects of TiAlN coating on tool life were different under various cooling conditions. The sequence for the improvement effects of TiAlN coating deposition under dry and the selected three cooling conditions was CSJC > FC > dry > MQL. It was illustrated that the low-temperature micro-droplets of fluid lubricant LN6889 could easily dissipate into the TiAlN tool–chip–workpiece contact interface compared to the micro-droplets of animal oil in the high-speed turning process of Ti-6Al-4V alloys.

## 5. Conclusions

In this research, a comparison of surface morphology and tool wear in the machining of Ti-6Al-4V alloys with uncoated and TiAlN tools under dry and cooling conditions was carried out. Several conclusions have been summarized as the following:

(1) The TiAlN coatings could not improve the average tool–chip contact friction characteristics in the initial dry turning Ti-6Al-4V compared to that achieved by uncoated tools. No improvement in cutting forces was found in machining Ti-6Al-4V with TiAlN tools compared to uncoated tools at a low speed of 75 m/min under CF, CSJC, and MQL cooling conditions. The TiAlN coating decreased 8.22%, 12.82%, and 12.50% of the tool–chip contact friction coefficients in turning Ti-6Al-4V at a high speed of 150 m/min under CF, CSJC, and MQL cooling conditions compared to that with uncoated tools, respectively.

(2) Superior surface roughness Sa was obtained in turning the Ti-6Al-4V alloy with uncoated and TiAlN tools under CF, CSJC, and MQL cooling conditions compared to that under dry cutting conditions. MQL and FC were the suitable cooling conditions in turning Ti-6Al-4V with uncoated and TiAlN tools by obtaining the lowest surface roughness. The improvement of MQL on machined surface roughness in turning Ti-6Al-4V with the TiAlN tool was similar to that under flood cooling conditions at a high speed of 150 m/min.

(3) The deposited TiAlN coating decreased the machined surface roughness by 4.09% and 0.74% in turning the Ti-6Al-4V alloy at cutting speeds of 75 m/min and 150 m/min under cryogenic spray jet cooling conditions. The deposited TiAlN coating decreased the machined surface roughness by 12.35% in the turning Ti-6Al-4V alloy at a low speed of 75 m/min under flood cooling conditions. However, the deposited TiAlN coating could not exhibit better improvements in machined surface roughness for the other cutting couples.

(4) The TiAlN-coated tools exhibited good antifriction effects in the initial turning of Ti-6Al-4V at a high speed of 150 m/min compared to that achieved by uncoated tools. The deposited TiAlN coating improved the tool life by 9.23%, 12.92%, 13.72%, and 2.05% in high-speed turning Ti-6Al-4V at a high speed of 150 m/min under dry, flood cooling, CSJC, and MQL conditions compared to that achieved by uncoated carbide tools, respectively. The improvement effects of the TiAlN coating on tool life were different under various cooling conditions. The sequence for the improvement effects of TiAlN coating deposition under dry and the selected three cooling conditions was CSJC > FC > dry > MQL.

In summary, the TiAlN coating could not significantly improve the machined surface roughness and tool wear of the cutting titanium alloy at a low speed of 75 m/min compared to that of the uncoated tools. TiAlN coating exhibited good tool life in turning Ti-6Al-4V at a high speed of 150 m/min compared to that achieved by uncoated tools. From the perspective of obtaining good, machined surface roughness and superior tool life, the TiAlN tool was one good selection in the high-speed turning of Ti-6Al-4V under the cryogenic spray jet cooling condition.

## Figures and Tables

**Table 1:** Chemical element composition of the Ti-6Al-4V alloy.

Elements	Ti	Al	V	Fe	Si	C	N	H	O	Other
Wt. %	base	5.5~6.8	3.5~4.5	≤0.30	≤0.15	≤0.10	≤0.05	≤0.0125	≤0.20	≤0.40

**Table 2:** Mechanical physical properties of the Ti-6Al-4V alloy at room temperature.

Properties	Tensile Strength	Proof Strength, Plastic Extension	Percentage Elongation after Fracture	Percentage Reduction of Area
Values	890 MPa	830 MPa	10%	25%

**Table 3:** Mechanical physical properties of the Ti-6Al-4V alloy at an evaluated temperature of 400 °C.

Properties	Tensile Strength	Percentage Elongation after Fracture	Percentage Reduction of Area	Long-Lasting Strength
Values	620 MPa	12%	40%	580 MPa

**Table 4:** Dry and various cooling conditions utilized in the turning process of Ti-6Al-4V.

Cooling Condition	Coolant Type	Supply	Instrument Type
Dry cutting	--	--	--
Flood cooling with cutting fluid (FC)	Water-soluble cutting fluid LN6889	--	--
Cryogenic spray jet cooling (CSJC)	Water-soluble cutting fluid LN6889	Air: 6–8 bar temperature: 0 °C	SM4000
Minimum quantity lubrication (MQL)	Animal oil	Air: 6–8 bar lubricant: 200 mL/h	SUNAIR PMP

## Data Availability

No data was used for the research described in the article.

## References

[1] Ezugwu E.O., Wang Z.M. (1997). Titanium alloys and their machinability—A review. J. Mater. Process. Technol..

[2] Rotella G., Umbrello D. (2014). Finite element modeling of microstructural changes in dry and cryogenic cutting of *Ti-6Al-4V* alloy. CIRP Ann. Manuf. Technol..

[3] Zhang S.J., To S., Wang S.J., Zhu Z.W. (2015). A review of surface roughness generation in ultra-precision machining. Int. J. Mach. Tools Manuf..

[4] Ulutan D., Ozel T. (2011). Machining induced surface integrity in titanium and nickel alloys: A review. Int. J. Mach. Tools Manuf..

[5] Pervaiz S., Deiab I., Darras B. (2013). Power consumption and tool wear assessment when machining titanium alloys. Int. J. Precis. Eng. Manuf..

[6] Chetan B.C., Behera S., Ghosh P.R. (2016). Wear behavior of PVD TiN coated carbide inserts during machining of Nimonic 90 and *Ti-6Al-4V* superalloys under dry and MQL conditions. Ceram. Int..

[7] Choudhary A., Paul S. (2019). Performance evaluation of PVD TiAlN coated carbide tools vis-à-vis uncoated carbide tool in turning of titanium alloy (Ti-6Al-4V) by simultaneous minimization of cutting energy, dimensional deviation and tool wear. Mach. Sci. Technol..

[8] Jaffery S.H.I., Mativenga P.T. (2012). Wear mechanisms analysis for turning Ti-6Al-4V-towards the development of suitable tool coatings. Int. J. Adv. Manuf. Technol..

[9] Zeilmann R.P., Weingaertner W.L. (2006). Analysis of temperature during drilling of Ti-6Al-4V with minimal quantity of lubricant. J. Mater. Process. Technol..

[10] Hao G.C., Liu Z.Q., Liang X.L., Zhao J.F. (2019). Influences of TiAlN coating on cutting temperature during orthogonal machining H13 hardened steel. Coatings.

[11] Hao G.C., Liu Z.Q. (2020). Thermal contact resistance enhancement with aluminum oxide layer generated on TiAlN-coated tool and its effect on cutting performance for H13 hardened steel. Surf. Coat. Technol..

[12] Nandgaonkar S., Gupta T.V.K., Joshi S. (2016). Effect of water oil mist spray (WOMS) cooling on drilling of Ti-6Al-4V alloy using ester oil based cutting fluid. Procedia Manuf..

[13] Strano M., Albertelli P., Chiappini E., Tirelli S. (2015). Wear behaviour of PVD coated and cryogenically treated tools for Ti-6Al-4V turning. Int. J. Mater. Form..

[14] Liang X.L., Liu Z.Q., Liu W.T., Li X.J. (2019). Sustainability assessment of dry turning Ti-6Al-4V employing uncoated cemented carbide tools as clean manufacturing process. J. Clean. Prod..

[15] Sterle L., Krajnik P., Pušavec F. (2021). The effects of liquid-CO_2_ cooling, MQL and cutting parameters on drilling performance. CIRP Ann..

[16] Ravi S., Kumar M.P. (2011). Experimental investigations on cryogenic cooling by liquid nitrogen in the end milling of hardened steel. Cryogenics.

[17] Gill S.S., Singh J., Singh R., Singh H. (2011). Metallurgical principles of cryogenically treated tool steels—A review on the current state of science. Int. J. Adv. Manuf. Technol..

[18] Dong J., Yu M., Wang W., Song H., Li C., Pan X. (2017). Experimental investigation on low-temperature thermal energy driven steam ejector refrigeration system for cooling application. Appl. Therm. Eng..

[19] Kalyan Kumar K.V.B.S., Choudhury S.K. (2008). Investigation of tool wear and cutting force in cryogenic machining using design of experiments. J. Mater. Process. Technol..

[20] Huang Y., Liang S.Y. (2003). Cutting forces modeling considering the effect of tool thermal property—Application to CBN hard turning. Int. J. Mach. Tools Manuf..

[21] Grzesik W., Denkena B., Zak K., Grove T., Bergmann B. (2016). Energy consumption characterization in precision hard machining using CBN cutting tools. Int. J. Adv. Manuf. Technol..

[22] Zorev N.N. (1966). Metal Cutting Mechanics.

[23] Braham-Bouchnak T., Germain G., Morel A., Furet B. (2015). Influence of high-pressure coolant assistance on the machinability of the titanium alloy Ti555–3. Mach. Sci. Technol..

[24] Chowdhury M.A., Das S., Debnath U.K. (2018). Estimation of the friction coefficient in turning process of metals through model experiment. Proc. Inst. Mech. Eng. Part J J. Eng. Tribol..

